# A neural network‐based framework to understand the type 2 diabetes‐related alteration of the human gut microbiome

**DOI:** 10.1002/imt2.20

**Published:** 2022-05-05

**Authors:** Shun Guo, Haoran Zhang, Yunmeng Chu, Qingshan Jiang, Yingfei Ma

**Affiliations:** ^1^ Shenzhen Institute of Synthetic Biology, Shenzhen Institutes of Advanced Technology Chinese Academy of Sciences Shenzhen Guangdong China; ^2^ Key Laboratory of Quantitative Engineering Biology, Shenzhen Institutes of Advanced Technology Chinese Academy of Sciences Shenzhen Guangdong China; ^3^ Shenzhen Key Laboratory of Synthetic Genomics; Guangdong Provincial Key Laboratory of Synthetic Genomics, Shenzhen Institutes of Advanced Technology Chinese Academy of Sciences Shenzhen Guangdong China; ^4^ Shenzhen Key Lab for High Performance Data Mining, Shenzhen Institutes of Advanced Technology Chinese Academy of Sciences Shenzhen Guangdong China

**Keywords:** human gut microbiota, neural network, random forest, T2D‐related microbial markers

## Abstract

The identification of microbial markers adequate to delineate the disease‐related microbiome alterations from the complex human gut microbiota is of great interest. Here, we develop a framework combining neural network (NN) and random forest, resulting in 40 marker species and 90 marker genes identified from the metagenomic data set (185 healthy and 183 type 2 diabetes [T2D] samples), respectively. In terms of these markers, the NN model obtained higher accuracy in classifying the T2D‐related samples than other methods; the interaction network analyses identified the key species and functional modules; the regression analysis determined that fasting blood glucose is the most significant factor (*p* < 0.05) in the T2D‐related alteration of the human gut microbiome. We also observed that those marker species varied little across the case and control samples greatly shift in the different stages of the T2D development, suggestive of their important roles in the T2D‐related microbiome alteration. Our study provides a new way of identifying the disease‐related biomarkers and analyzing the role they may play in the development of the disease.

## Highlights

- A framework combining neural network and random forest for identifying the type 2 diabetes (T2D)‐related biomarkers.
- Constructing the directed interaction networks of the biomarkers for analyzing the potential drivers of the microbial community associated with T2D.
- Analyzing the covary of the biomarkers with the dynamic change of fasting blood glucose in the development of T2D.

## Graphical abstract

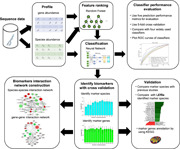

## INTRODUCTION

The gut microbiome, which inhabits the human intestinal tract, is a complex ecosystem consisting of 10^14^ microbial cells [1] and has been identified as playing a central role in human health and a variety of diseases [2–4]. With the advent of the next‐generation sequencing technology, overwhelming amounts of microbial metagenomic sequencing data generated from human gut samples have been obtained. Metagenomic studies have provided great opportunities to get valuable insights into how the gut microbiota is associated with various human diseases via various well‐developed bioinformatic tools and algorithms. In the field of microbiome research, a common practice is to utilize statistics‐based strategies (e.g., Spearman's correlation coefficient, Wilcoxon rank‐sum test, etc.) to identify the biomarkers associated with the gut microbiome according to the disease states [5–10], such as the widely applied software LEfSe [5, 11, 12]. These methods can identify metagenomic features that have statistically significant differences between case and control groups. Nevertheless, these statistics‐based analyses are typically based on the independent or linear assumption, whereas dysbiosis of intestinal flora is complex and likely depends on the nonlinear effects of many microbes [13]. To this end, these methods may neglect some potential metagenomic biomarkers that might contribute to the disease‐related alterations of the human gut microbiome but have no detectable statistic changes across the samples. For example, *Veillonella parvula* has been reported to be related to type 2 diabetes (T2D) [14, 15], however, the abundance of which varied little across the samples [6].

Machine learning has recently attracted growing attention in biological research. Some well‐known algorithms including Support Vector Machine (SVM), Random Forest (RF), Hidden Markov Model (HMM), Bayesian network (BN) as well as Gaussian network, have been applied in the prediction of the protein binding [16], the metabolic functions in microbial communities [17], characterization of the transcriptional networks [18], and so forth. As machine learning can generate models and find predictive patterns from large datasets, it would impact microbiome research and other biology fields [19]. However, these traditional machine learning algorithms have some limitations. For example, SVM is a linear model and HMM and BN depend on some probability‐based hypotheses. To this end, some T2D‐related species, such as *Coprobacillus catus* [14, 15] would not be identified by these methods since the abundance of the species varies little across samples (may not have a linear correlation with the disease) and the averaged abundance is very low (would be the outlier under some probability distribution) [6]. Deep learning‐based methods, which typically use the neural network (NN) with multiple hidden layers, show promising performance in recent studies as they can identify novel patterns that would have been ignored by other methods in a given complex data set [20]. Recently, these methods have been applied in the field of biology, such as predicting special genes/protein functions [21, 22], identifying medical diagnoses [23], drug discovery [24], and so forth. However, one limitation of appling the technology in the microbiome‐related field would be that the number of available samples in these studies is typically limited (e.g., several 100 samples). As for the deep learning model, there are usually many layers and neurons (determine the size of the parameters in the model), which require massive samples for training. Besides that, applying the NN model to the microbiome data may remain a concern due to its “black box” nature, that is, it is often difficult to demonstrate which input feature plays a decisive role in the output.

Here, we propose a framework combining NN and RF algorithms for identifying the biomarkers in linking the gut microbiome with T2D based on the microbial profiles. To demonstrate the utility of our approach, we took advantage of two publicly available independent metagenomic datasets from Chinese diabetes patients and nondiabetic controls [6]. According to the identified markers, we first demonstrated that fasting blood glucose (FBG) is the most important factor associated with the T2D‐related alteration of the human gut microbiome and uncovered that those microbial markers, which vary little across the case and control samples, might also play an important role. The results of our analyses suggest that the cumulative effect of these marker species rather than individual species likely drives the T2D‐related alterations of the human gut microbiome. This study paves a way to use the NN algorithm in microbiome study and provides potential opportunities to deeply understand the microbial roles in the development of human diseases and evaluate individuals at risk of relevant diseases.

## RESULTS

### The NN model performs better than other methods

**Figure 1:**
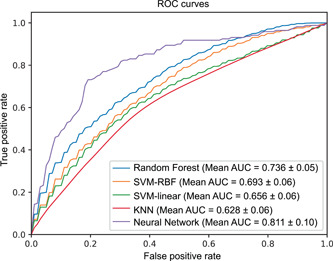
Cross‐validation analysis for T2D discrimination on the D1 data set. The receiver operating characteristic (ROC) curve of five classifiers (RF, SVM‐linear, SVM‐RBF, KNN, Neural Network) was obtained by using fivefold cross‐validation. AUC, area under the curve; RBF, radial basis function; KNN, K‐nearest neighbor; RF, Random Forest; SVM, Support Vector Machine

For comparison, we accessed the prediction performances of the NN model as well as SVM, SVM‐radial basis function (RBF), RF, and K‐nearest neighbor (KNN) methods in classifying the T2D‐related samples of the D1 data set based on the microbial profile using fivefold cross‐validation (CV). The obtained results of the five methods with all metrics were listed in Table S8. The receiver operating characteristic (ROC) curves were plotted in Figure 1 and thus the corresponding area under the curve (AUC) metrics were calculated for each method. Clearly, compared with the other four methods, the NN model performed best for all metrics (AUC: ∼0.8, other metrics: ∼0.75). This may due to the nonlinear fitting ability of the NN model, and it would extract more predictive features. As for the other four methods, two nonlinear classifiers, that is, RF (AUC: 0.736 ± 0.05) and SVM‐RBF (AUC: 0.693 ± 0.06) performed better than the rest two ones (SVM AUC: 0.656 ± 0.06, KNN AUC: 0.628 ± 0.06).

### The identified marker species play a decisive role in the T2D‐related alteration of the human gut microbiota

**Figure 2:**
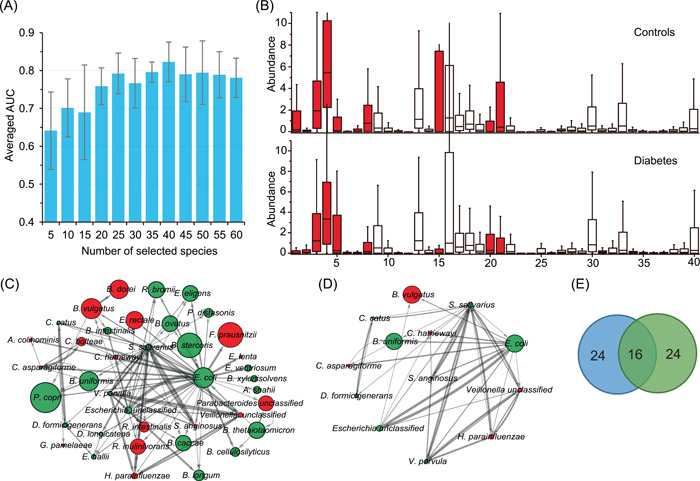
Marker species identification and interaction network construction. (A) Averaged area under the curve (AUC) of classification with a different number of the ranked species based on Neural Network‐based classifier by using fivefold cross‐validation on the species‐level profile of D1. And the peak value could be obtained as the 40 species were selected, thus these species were identified as the marker species in this study. (B) The distribution of the abundance of marker species in diabetes‐related dysbiosis. A comparison between the cases (*n* = 183) and controls (*n* = 185) from the species‐level profile of D1. Using Kruskal–Wallis test, the species with red color were identified that the abundances varied significantly (*p* < 0.05) across all samples. The order of the marker species is the same as that in Table S1. (C) The interaction network of marker species was determined by GENIE3, where the top 100 most reliable interactions between marker species were selected. In the network, each node represents the respective marker species; the constructed internet work is directed and the arrow between nodes indicates the direction of the interaction, namely, each arrow from Node A to B means that the abundance of B is mainly affected by that of A. The width of the edges between nodes is proportional to the reliability of the linkage between the nodes. The size of each node is proportional to the averaged abundance of the maker species. The network layout was visualized by the Cytoscape software using a circular layout. The nodes with red color are the marker species that significantly are enriched in the diabetic samples or the control samples. (D) Interactions between marker species with a degree more than 5. (E) The Venn diagram of the number of marker species selected by our method and LEfSe (see Table S2)

We further investigated which species rather than all species of the gut microbiota, play a decisive role in classifying the T2D‐related samples using the NN model. Due to the “black box” nature of the NN model, therefore, we used a widely employed feature selection method‐RF [25] for ranking all species of D1 samples according to their importance scores (default metric: mean decrease accuracy). We then fed top *k* (*k* = 5, 10, 15, 20, …, 60) species to the NN model respectively for classifying the samples. The prediction performances of the NN model using different numbers of species were accessed according to their average AUC of fivefold CV in classifying the T2D‐related samples. It can be seen from Figure 2A that the NN model reaches the peak average AUC value (*82.3* ±* 5**%***) with the top 40 species. This prediction result is even slightly better than that of using all species (*n* = 270) (one reason may be that there are some noises in all these species, thus it would impact the prediction performance), suggesting that the selected marker species can be used to delineate the T2D‐related alteration of the gut microbiota to some extent. Therefore, we took the selected top 40 species as the marker species (Table S1).

To evaluate our selected marker species, we chose the widely applied software LEfSe [5] to identify the marker species, coincidentally, resulting in 40 markers (|linear discriminant analysis) scores|>2) associated with T2D (Figure S1). Especially, 16 of these 40 marker species are shared by the NN model and LEfSe while the other 24 marker species are different (Table S2). Additionally, we also used the software *ANCOM‐II* [26] to check our results. Interestingly, ANCOM‐II identified 19 species (cutoff: 0.7, see Table S7), while *17 out of 19* are contained in our selected biomarkers. Then, we compared the prediction performance of the five classifiers with the three different marker species using a fivefold CV on the D1 data set (see Table S9). As a result, all classifiers with our selected marker species performed better than those with LEfSe biomarkers (with the AUC improvement ∼1% – ∼5%), while two of them (i.e., KNN and SVM‐RBF) performed worse than that with ANCOM‐II biomarkers. One possible reason may be that the algorithm of LEfSe ignores some important biomarkers with discriminant ability, the abundances of which show little changes across the case and control samples.

Furthermore, we employed the independent data set D2+ to assess the prediction capability of the NN model with the 40 marker species. In this test analysis, with these marker species, the species‐level profile of D1− was utilized for training while that of D2+ was used for testing and the AUC in classifying T2D samples of D2+ reached *76.2%*.

To characterize the variations of the 40 marker species identified by the NN model across the D1 samples, we compared the distribution of their relative abundances in case and control samples (Figure 2B). Based on the result obtained using the Kruskal–Wallis test, we can observe that only 16 of 40 marker species vary significantly between the case and control samples in their relative abundances (*p* < 0.05). However, some of the marker species vary little across the case and control samples (e.g., *Ruminococcus bromii* [ID: 40], *C. catus* [ID: 37], and *Collinsella aerofaciens* [ID: 38]) have been detected associated with T2D in previous studies [27, 28]. This observation suggests that the marker species that do not vary significantly across the case and control samples likely play a special role in the gut microbial alteration related to T2D.

### The interaction network identifies the key species in the alteration of the human gut microbiome

To further investigate the relationship among these marker species, we constructed the species–species interaction network based on the vector of the relative abundances of the marker species of D1. The widely applied GENIE3 [27] was implemented, which calculates the interaction by considering the nonlinear relationship of multiple species. In this experiment, all possible interactions between species–species were ranked according to their reliability scores calculated by GENIE3, and we first selected the top 100 interactions for constructing the interaction network. As shown in Figure 2C, in total, 39 of the 40 marker species have at least one connection with others, 13 species connect with at least five other species (Figure 2D), and four species, including *V. parvula*, *Streptococcus salivarius*, *Escherichia coli*, and *Escherichia_unclassified*, have more than 10 connections.

Moreover, the relative abundances of seven (green color) of the thirteen species do not vary significantly across the case and control samples (Figure 2D). Among the seven species, *E. coli* and *S. salivarius* have the highest number of connections, 35 and 25, respectively, with the rest species, suggesting the importance of these two species in the T2D‐related alteration of the human gut microbiome. *E. coli* has been identified to be diabetes‐enriched [6] and *S. salivarius* can ferment glucose yielding lactic acid [29]. Nearly 70% (*n* = 9) of the 13 marker species of (Figure 2D) are short‐chain fatty acids‐producing bacteria and previous studies have demonstrated that these types of bacteria played an important role in T2D [6, 14].

Additionally, among the 13 species, except for *E. coli*, *Bacteroides vulgatus*, and *Bacteroides uniformis*, other species are lowly abundant in the human gut samples. Thus, these findings from the interaction network analysis based on the selected marker species suggest that the species with low abundance may also play a critical role in the T2D‐related alteration of the human gut microbiome through interacting with other species. Therefore, the marker species (*n* = 13) with interactions of more than five (Figure 2D) can be assigned as the core of the gut microbiota associated with T2D.

### The identified marker genes are responsible for the T2D‐related alterations of gut microbiota

**Figure 3:**
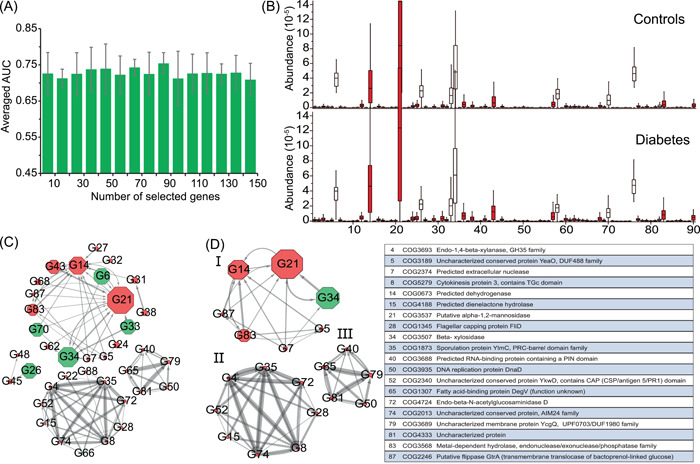
Marker gene identification and interaction network construction. (A) Averaged area under the curve (AUC) of classification with the different number of the ranked genes based on Neural Network‐based classifier by using fivefold cross‐validation on the gene profile of D1. And the peak value could be obtained as the 90 species were selected, thus these genes were identified as the marker genes in this study. (B) The distribution of the abundance of the marker genes significantly varied across samples in diabetes‐related dysbiosis. A comparison between the cases (*n* = 183) and controls (*n* = 185) from the gene profile of D1. Using Kruskal–Wallis test, the species with red color were identified that the abundances varied significantly (*p* < 0.05) across all samples. The order of the marker genes is the same as that in Table S6. (C) The interaction network of marker genes was determined by GENIE3, where the top 100 most reliable interactions between marker genes were selected. The width of the edges between nodes is proportional to the reliability of the linkage between the nodes. The size of each node is proportional to the averaged abundance of the marker genes. The network layout was calculated by the Cytoscape software using a circular layout. The number in each node is the ID of the marker species, which is consistent with Table S3. The nodes with red color are the marker species that significantly enriched in the diabetic samples or the control samples. (D) Interactions between marker genes with a degree of more than five

To identify the T2D‐associated gut metagenomic markers on both genetic and functional levels, we also applied our method to the functional gene profile of D1. Similarly, we ranked the genes using the RF method and then evaluated the average AUC of the NN model with different ranked genes using a fivefold CV. As seen in Figure 3A, the peak value (*75.4* ± *3%*) of the average AUC was obtained when the top 90 genes were selected for classification. To this end, we took the 90 genes as the marker genes (Table S3).

We further tested the NN model using the 90‐marker genes (trained on the D1− data set) in identifying the T2D samples of the D2+ data set based on the gene profile, resulting in 74.6% AUC. In the same way, we compared the relative abundances of these marker genes in the case and control samples of the D1 data set and observed that 78 out of the 90 genes vary significantly (*p* < 0.05) using Kruskal–Wallis test (Figure 3B).

### The interaction network of the marker genes identifies core functional gene modules related to T2D

To characterize the interactions between the marker genes, we also constructed the gene–gene interaction network using GENIE3 (Figure 3C), resulting in 37 of 90 marker genes having at least one connection with other genes. As seen in Figure 3C, the interaction network of the maker genes is partitioned into four separate groups and the genes connect with others within each group. Few genes (*n* = 20) have more than five connections with other genes (Figure 3D). We utilized BlastKOALA for annotation and Kyoto Encyclopedia of Genes and Genomes (KEGG) mapping to characterize the functional category of the 90 marker genes in functional modules or pathways. As a result, we find that 56.7% (*n* = 51) of the marker genes can be annotated in the database Clusters of Orthologous Groups (COGs; Figure S2). Only 16 of 90 genes can be mapped to the functions in the KEGG database. According to the functional annotations of these marker genes in the KEGG database, 4 of 18 genes in Group I (ID:34(COG3507), 70(COG2160), 87(COG2246), 68(COG1071)), 2 of 3 genes in Group II (ID: 48(COG 1211), 26(COG0493)), 1 of 11 genes in Group III (ID:66(COG2971) were mapped to the pathways of carbohydrate metabolism (Table S4), suggesting these genes in the groups are likely involved in or associated with the carbohydrate metabolism. One of eleven genes in Group III (ID: 28(COG 1345) was mapped to flagellar assembly. The maker genes connect with others in each group but do not interact with those in other groups, implying the genes in different groups function differently in carbohydrate metabolism. The interaction of these genes confirms the link between the T2D‐related alterations of the human gut microbiome and carbohydrate metabolism [14, 15, 30].

### NN‐based regression analysis uncovers the most significant covariate associated with the T2D‐related alteration of the human gut microbiome

**Figure 4:**
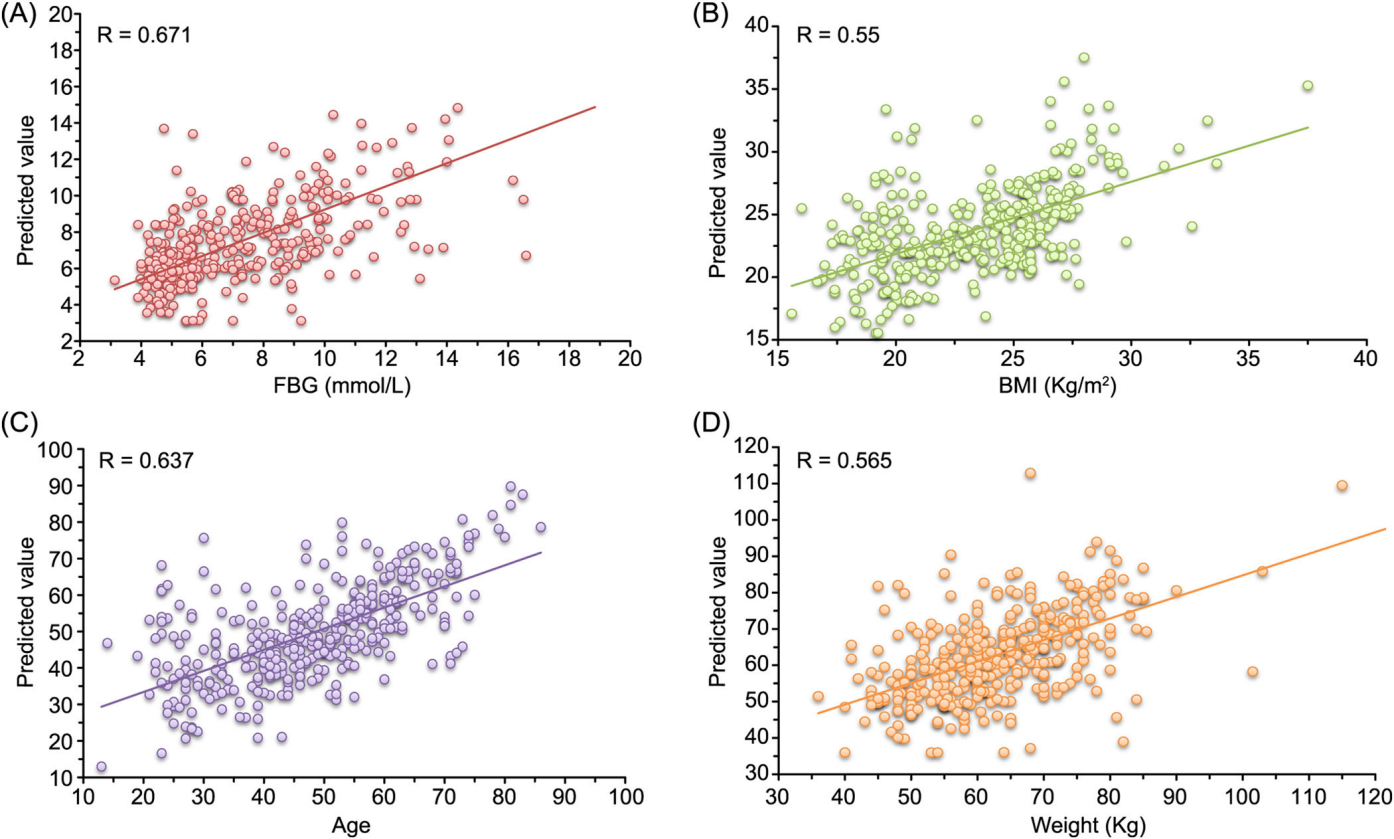
Neural Network‐based predicting values and the actual values of T2D‐related factors based on the species level profile of D1 with our marker species. (A) FBG, (B) BMI, (C) age, and (D) weight. Each T2D‐related factor value was fit by the corresponding NN‐based regression model using fivefold cross‐validation, and the *R*‐value (i.e., Pearson's linear correlation coefficient) between the real values and the predicted values was obtained by statistical calculation. BMI, body mass index; FBG, fasting blood glucose; T2D, type 2 diabetes

FBG, age, body mass index (BMI), and the weight of patients all potentially are causative factors of T2D. To determine the most significant factors associated with the human gut microbiome represented by the selected microbial markers, we did the regression analysis of D1 with the 40 marker species. Based on the fivefold CV, for each factor, the predicted values of the samples were calculated by the NN model. Then, these values and the corresponding real values were plotted (Figure 4). These figures demonstrate that for each factor, the microbiome values predicted based on the markers are linearly correlated with the corresponding real values across the samples (Figure 4A–D), suggesting that the human gut microbiome covaries with these factors. Particularly, based on the observation that the microbiome correlation with FBG (*p* < e−50) is more significant than with other factors (age, e‐43; BMI, e‐30; weight, e‐32), we can conclude that FBG is likely the leading factor in driving the T2D‐related alteration of the human gut microbiome in the development of T2D. Although several works [28, 31] have reported the relationship between the gut microbiome and FBG, our result reveals the strong correlation between FBG and the T2D‐related alteration of the human gut microbiome using nonlinear regression rather than classification.

**Figure 5:**
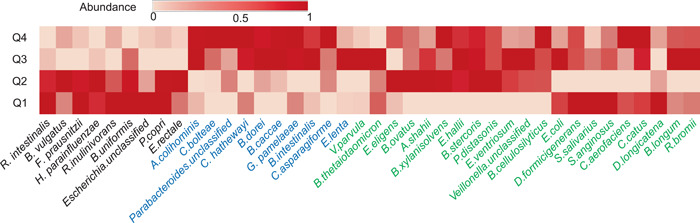
Heat map of mean relative abundance of the 40 selected marker species for the four dynamic intervals of fasting blood glucose. The dynamic intervals were determined by the quantile statistics (Q1: < 5.02; Q2: 5.02–6.21; Q3: 6.21–8.8; Q4: > 8.8). In each dynamic interval, the abundances of marker species for the corresponding samples were averaged. Then, the averaged abundances of each marker species for the four dynamic intervals were normalized (mapping to [0, 1]). The species names were colored based on the significance analysis across samples using Kruskal–Wallis test (black: abundances significantly increase in healthy samples; blue: abundances significantly increase in T2D samples; green: abundances have no significant differences between T2D and healthy samples). T2D, type 2 diabetes

To explore how our marker species co‐vary with the dynamic change of FBG in the development of T2D, we plotted the heat map of mean relative abundances of the marker species at four intervals (i.e., Q1: < 5.02; Q2: 5.02–6.21; Q3: 6.21–8.8; Q4: > 8.8) of FBG (Figure 5). It can be observed that the relative abundances of these marker species vary greatly at different intervals (Figure 5). Among the 16 marker species with a significant difference in relative abundance between case and control samples (*p* < 0.05) based on the Kruskal–Wallis test, six have higher relative abundances in healthy control samples, 10 in T2D samples. Those six marker species (Figure 5, black) are higher abundant in both Q1 and Q2 than in Q3 and Q4 samples. Among those 10 maker species, six are higher abundant in both Q3 and Q4, 1 (*Bacteroides dorei*) in Q4, one (*Eggerthella lenta*) in Q3, and two (*Veillonella. unclassified* and *Streptococcus anginosus*) in both Q1 and Q3. These marker species are identified as T2D‐related species easily according to their relative abundances in T2D (high FBG) and healthy samples (low FBG) using conventional statistic‐based methods. Other 24 marker species that do not have significant differences in relative abundance between the case and control samples exhibit diverse patterns. Intuitively, some of them (*n* = 3, *Dorea longicatena*, *Prevotella copri*, and *S. salivarius*) reach the highest abundance in Q1, two (*Bacteroides ovatus*, *Escherichia.unclassified*) in Q2, five (*V. parvula*, *Bacteroides xylanisolvens*, *Eubacterium eligens*, *R. bromii*, *Bifidobacterium longum*) in Q3, three (*Alistipes shahii*, *E. coli*, and *Ensete ventriosurn*) in both Q2 and Q3, two (*Bacteroides thetaiotaomicron*, *Eubacterium hallii*) in both Q2 and Q4. Thus, these 24 marker species vary in different patterns associated with the dynamic changes of FBG. This may explain the reason that the two important T2D‐related marker species (*E. coli and S. salivarius*) detected by the interaction network analysis vary little across the case and control samples. In conclusion, our analysis suggests that these marker species are likely to play a different role in different stages of T2D development and then the cumulative effects of these markers drive the T2D‐related alteration of the human gut microbiome.

## DISCUSSION

To provide new perspectives for understanding the roles of the gut microbiota in the development of T2D, here we present the NN‐based framework to identify microbial markers that can be used as representatives of the gut microbiota for the prediction of T2D‐related samples with relatively high performance. A number of markers not detectable by conventional statistic methods vary little across the case and control samples but interaction network analysis and regression analysis indicates that they likely play a crucial role in the T2D‐related alteration of the human gut microbiome as well. This framework firstly uncovers the T2D‐related dynamics and interaction of the microbial markers in the human gut microbiome, strongly suggesting the cumulative effect of the markers is likely the driver of the gut microbiome alteration.

The deep learning approach usually requires massive samples for training and feature extraction. Although numerous T2D‐related human gut microbiome studies have been completed [32], we only recruited the datasets generated by whole genomic sequencing and are thus eligible for this study [6, 15]. The main limitation of our study is that only a small number of samples (*N* = 368, D1) were recruited for training the NN model. Even so, by determining the suitable numbers of layers and nodes, our model obtained relatively higher performance in classifying the T2D‐related samples in the CV experiment than other approaches. Due to the lack of control samples (healthy subjects) in the data set D2, we set up the data set D2+ comprising 30% control samples of D1 that were randomly selected as the control and the samples of D2 as the case. When we applied the framework on D+ using the marker species, the prediction AUC of the model reached 76.2%, slightly lower than the result of the fivefold CV experiment.

The classification results demonstrate that our NN model performs better than other conventional methods including SVM‐linear, SVM‐RBF, RF, and KNN (Figure 1), showing its relative high capability to uncover novel patterns in complex microbiome datasets. Nevertheless, applying the NN model to the microbiome data is still challenging due to the “black box” nature of it, i.e. it is often difficult to interpret which input features play a decisive role to the output. To overcome this limitation, in this study, the importance of the features (i.e., species or genes) in distinguishing the case and control samples was calculated by RF, and subsequently, the NN model as the classifier was used for determining which subset of the features (ranked with the importance) was the most important according to the prediction performance of the classifier with the corresponding subset. Our analysis resulted in 40 marker species and 90 maker genes, respectively. The NN model using these markers to predict T2D‐related human gut samples obtained slightly better performance than that of using all the microbial features. Interestingly, most of our marker species could be assigned to the known bacterial species, which had been reported to be associated with diabetes in previous studies [6, 33, 34] (Table S6). For example, the marker species *C. catus* has been reported to be the producer of short‐chain fatty acids (e.g., propionate and butyrate) [14, 35], while the studies in Zhao et al. [15] demonstrated that deficiency in short‐chain fatty acid production was related to T2D. The abundance of *R. bromii* was observed to be reduced from controls to chronic pancreatitis (CP) nondiabetics to CP diabetics in Jandhyala et al. [36]. In Dewulf et al. [37], the researchers found that the increased level of *C. aerofaciens* could be a beneficial effect associated with inulin‐type fructans fermentation, which might be used to control related metabolic disorders including diabetes.

Our analysis demonstrates that all our experimental classifiers with our selected markers performed better than those with the markers identified by *LEfSe*. When we analyzed the abundance distributions of the selected biomarkers across the case and control samples using the Kruskal–Wallis test, quite a part of our marker species (*n* = 24) varied little in their abundances. Thus, we hypothesize that the interactions of the marker species, rather than individual species, affect the T2D‐related microbiome. To address this, we constructed the directed biomarker interaction networks and the networks provide the orientation information on how the biomarkers impact each other (Figures 2C,D and 3C,D). These biomarker interactions demonstrate the patterns of the microbial marker species interfering with each other. For example, our marker species interaction network indicates that *E. coli* and *S. salivarius* impact almost all other marker species, suggesting that they are likely the main drivers of the network. Meanwhile, *E. coli* is impacted by *B. thetaiotaomicron* and *S. salivarius*; thus, *B. thetaiotaomicron* could be considered as the potential indirect driver of the microbial community. The interactions among these marker species may carry more information than the individual species. In this regard, the marker species that do not vary significantly in relative abundance across the case and control samples likely play a special role in the gut microbial alteration related to T2D. The Kruskal–Wallis test is based on the independence assumption, thus it would not take the interaction between the species into account, while our method takes nontrivial relationships in the data into account and therefore will produce better answers.

Regression analyses show that the human gut microbiome represented by the selected microbial markers has a strong correlation with FBG as well as BMI, age, and the weight of the corresponding patients (*p* < 0.05). All these are likely the causative factors of T2D, but in terms of the *R* and *p* values, FBG (*R* = 0.671, *p* = 4.87e−51; Figure 4) is the most important factor associated with the human gut microbiome. This finding implies that the gut microbial composition of patients likely keeps altering with the increase of FBG in the development of T2D. We stratified the samples of D1 into four intervals according to their FBG values (i.e., Q1: < 5.02; Q2: 5.02–6.21; Q3: 6.21–8.8; Q4: > 8.8) and plotted the heat map of mean relative abundances of the marker species of the samples within each interval. We can observe that the 24 marker species that do not vary significantly across the case and control samples show different patterns in relative abundance across the four intervals of FBG. Our analysis indicates that these 24 marker species including *E. coli* and *S. salivarius*, greatly affect the performance of the NN model in classifying the T2D samples and are key species in the interaction networks. Because the FBG is gradually increasing in the development of T2D, the species altering with the increasing FBG are likely more important in driving the T2D‐related gut microbiome alteration than those with significant differences between case and control samples identified by statistic‐based methods. These results provide a different perspective for understanding the relationship between diabetes and the human gut microbiome and thus need further investigations.

## CONCLUSION

This study applies a framework combining NN and RF to reanalyze the human microbiome for identifying the T2D‐related microbial markers, and many of these markers are neglected by other statistic‐based approaches. In the validation and test analysis, these markers were used to predict the disease state of the samples and thus can be used to explain the microbial alterations of the human gut microbiota in the development of T2D. Construction of the directed interaction networks of the markers captures the potential drivers of the microbial community associated with the diseases, suggesting the complex nature of the human gut microbiota where many core species interactions instead of bacterial individuals impact the related disease. In conclusion, using the NN model and RF, our analysis yields new knowledge of the role of the human gut microbes in the development of T2D.

## METHODS

### Datasets

The whole metagenomic sequencing datasets used in this study were generated in two studies, respectively [6, 15]. The first data set D1 [6] (accession number in GenBank: PRJNA422434) is based on the shotgun sequencing of DNA extracted from the stool samples of Chinese individuals, where it contains 183 samples from the subjects with T2D and 185 control samples from healthy subjects. The second data set D2 [15] (accession numbers: PRJEB15179 and PRJEB14155) are obtained from the metagenomic shotgun sequencing of 391 samples from the Chinese patients with T2D at different days (0, 28, 56, and 84), where all samples are divided into two groups with two diets for studying the effects of the dietary on T2D. In this study, the data set D1 was used for analyzing the prediction performance via CV and selecting the microbial markers. As an independent data set, D2 was used for further evaluating the prediction performance with our selected microbial markers. However, since the data set D2 has only positive samples (i.e., case samples), 30% of control samples (*n* = 56) of D1 were randomly selected as the control for D2. The rest samples of D1 were assigned as data set D1−. D2 and the added control samples (i.e., D2+) can be viewed as the data set that is independent of D1−.

All metagenomic samples were profiled on taxonomic species and functional gene levels, respectively. We first calculated the relative abundances of the species for each sample resulting in 270 species for analysis. The abundances of these species were calculated for all samples of D2. The genes encoded by the metagenomes were assigned using the COG database and the relative abundances of 4632 genes present in at least two samples were calculated for each sample for profiling D1 at the gene level. The relative abundances of these genes were calculated in the D2 datasets as well.

### Taxonomic abundance and gene abundance calculation

The microbial profile of each sample at the taxonomic species level was estimated by the software MetaPhlAn2.7.4 [38] based on the metagenomic sequencing read data and the relative abundance of each species was calculated. The bacterial gene profile of each sample was estimated using the software Diamond (v0.9.14.115) against the COG (Clusters of Orthologous Groups) database with the cut‐off value of 1e−10 [39]. The genes present in at least two samples were calculated for each sample for profiling D1 at the gene level. The abundance of each gene was calculated based on the number of the metagenomic sequencing reads and normalized according to the total read number of the sample.

### NN model and the comparative classifiers

In this study, the datasets used for experiments contain only hundreds of samples. Thus, to reduce the scale of the model parameters, we designed a lightweight NN model with two dense hidden layers of 16 and 8 nodes using the “Rectified Linear Unit” as the activation function, respectively. Moreover, a l2 regularization was introduced for preventing overfitting. We chose the widely used cross‐entropy as the loss function and Adam as the optimizer. The hyper‐parameters of the model (e.g., the number of nodes in each layer, number of epochs, batch size, etc.) were tuned using fivefold cross‐validation based on the AUC metric. In fivefold CV, all samples of the data set are randomly divided into five equal size subsamples, four are used for training, and the remaining one for testing. The process was repeated five times, and each of the five subsamples was used once as the testing sample. The different values of each hyper‐parameter within the corresponding range were considered in the experiment and the one maximized the AUC was chosen as the final setting.

Meanwhile, the prediction performances of four widely used classifiers, including SVM‐linear, RF, SVM‐RBF, and KNN were also evaluated in the same way for comparison. All these classifiers were implemented on the microbial profile of D1 at the species level, and their prediction performance metrics were averaged by a fivefold CV in the experiment (see Figure 1).

### T2D‐related marker species identification and evaluation

We incorporated RF with the NN model to identify the marker species related to T2D. Briefly, the RF method was firstly implemented on the species‐level profile of D1 samples to calculate the scores (i.e., feature weights) of all the species according to their impacts on the classification results as random noise was added to them. In light of the scores, we ranked all the species of the samples in D1. Then, the feature selection process was implemented using the NN model on the ranked species‐level profile of D1. Using fivefold CV, the average AUC of the NN model with top‐*k* (*k* ranged from 5 to 60) species was calculated. The number of the species (i.e., *k*) that maximized the AUC was chosen as the optimal number and thus the corresponding species were selected as T2D‐related marker species contributing to the alteration of the human gut microbiome (see Figure 2A).

To further evaluate our marker species, the markers selected by the widely applied software LEfSe [5] were chosen as a baseline for comparison. Specifically, we analyzed the prediction performance (i.e., AUC) of the five classifiers with these two groups of marker species respectively using fivefold CV on the D1 data set (see Table S9).

Besides that, we also calculated the prediction performance of the NN model with our markers (training on the data set D1−) on an independent data set D2+.

### Marker species interaction network inference and analysis

To further study the relationship between the selected marker species, GENIE3 was utilized for constructing the species‐species interaction network using the marker species. The package of “GENIE3” of the MATLAB software was performed with default parameters in our experiments to calculate the scores of the links between the marker species according to the correlation strength, and then the links were ranked based on the scores from high to low. The higher the score is, the more reliable the link between the marker species is. We also applied a refinement procedure [40] to improve the constructed network under the assumption that the links of the hub nodes would be more important and reliable. Once GENIE3 was implemented, an adjacency matrix *M* will be obtained, where *M*_*ij*_ represents the reliability level of the link from node *i* to node *j*. And the refined adjacency matrix $\hat{M}$ is given as:

(1) $$\hat{M}(i,:)=M(i,:)\;*\;\sigma^{2},$$

where $\hat{M}\left(i,:\right)$ is the *i*‐th row of M and $\sigma^{2}$ is a variance in the *i*‐th row of M. It should be noted that if many links come from the same node, the variance in a row of M corresponding to the node would be elevated. Finally, we selected the top 100 links in our experiments to reconstruct the interaction network.

### Marker gene identification and analyzation

In the same way as species marker identification, we identified the marker genes by incorporating RF with the NN model based on the gene profile of the D1 data set. Briefly, in this experiment, after all the genes were ranked by RF, the average prediction AUC of the NN model using the gene profile of D1 with top *k* (*k* from 5 to 100) genes was calculated using a fivefold CV. Then, we selected the T2D‐related marker genes, with which the NN model has the highest average AUC in classifying T2D‐related samples.

Furthermore, we analyzed the potential functions of these marker genes by mapping them to the KEGG database. In addition, the prediction performance (i.e., AUC) of the NN model with these marker genes (trained on the data set D1−) in classifying T2D‐related samples was tested on the independent data set D2+.

### Marker gene interaction network inference and analysis

As the same procedure of the inference of the marker species interaction network, the gene‐gene interaction network was constructed by using GENIE3 based on the gene profile of the D1 data set with the marker genes. Similarly, we selected the top 100 links of genes to reconstruct the interaction network.

### NN‐based regression analysis of the human gut microbiome and the T2D‐related factors

To further investigate the correlations of the T2D‐related factors (including FBG, age, BMI, and body weight) with the T2D‐related alterations of the gut microbiota, the NN‐based regression analysis was performed. Specifically, the regression model for each factor was built using the similar structure of our NN‐based classification model (i.e., two hidden layers with the same number of nodes in these layers), while for the output layer, one node without using the activation function was applied for regression. We used the mean‐square error as the loss function for implementation. The values of each factor in D1 (i.e., the dependent variable) were predicted by the corresponding trained model with our marker species (the dependent variables) using a fivefold CV. Moreover, the correlation coefficient (i.e., Pearson's linear correlation coefficient), as well as the corresponding *p*‐value between the predicted values and the real values for each factor in D1 were calculated, which reflected the degree that the factor was influenced by the abundances of the marker species to some extent.

Moreover, to explore how the markers altered along with the dynamic change of FBG in the development of T2D, we stratified all samples of D1 into four intervals according to the quantile statistics of the corresponding FBG values (i.e., Q1: < 5.02; Q2: 5.02–6.21; Q3: 6.21–8.8; Q4: > 8.8). Then, the averaged abundances of the marker species of the samples within the corresponding interval were calculated and normalized (mapped to [0, 1]).

### Accuracy, precision, recall, and F1 metrics

The metrics were defined as follows:

(2) $$\begin{array}{l} \mathrm{accuracy}=\frac{\mathrm{TP}+\mathrm{TN}}{\mathrm{TP}+\mathrm{FP}+\mathrm{TN}+\mathrm{FN}},\\ \mathrm{precision}=\frac{\mathrm{TP}}{\mathrm{TP}+\mathrm{FP}},\\ \mathrm{recall}=\frac{\mathrm{TP}}{\mathrm{TP}+\mathrm{FN}},\\ F1=\frac{2\;*\;\mathrm{precision}\;*\;\mathrm{recall}}{\mathrm{precision}+\mathrm{recall}}, \end{array}$$

where TP indicates true positives (i.e., a case sample is predicted correctly), FP false positives (a control sample is predicted as a case sample), and FN false negatives (a case sample is predicted as a control sample). The accuracy measure reflects the overall quality of classification for the classifier. The precision measure focuses on how accurate the diabetic status predicted by the classifier is. The recall measure answers the question that what proportion of the diabetes samples could be identified by the classifier. And F1 measure is the integration of the precision measure and the recall measure.

### ROC curve and AUC metric

The ROC curve indicates the true positive rate (i.e., the number of correctly predicted case samples divided by the total number of samples predicted as case samples) against the false positive rate (i.e., the number of samples that are wrongly predicted as case samples divided by the total number of samples predicted as control samples). The AUC metric ranges from 0.5 to 1 (the greater, the better), and is computed from the ROC curve, which summarizes false positive and true positive rates. The AUC is robust to the unbalance situation of each outcome.

## AUTHOR CONTRIBUTIONS

Shun Guo did the experiments, analyzed data, and wrote the manuscript. Haoran Zhang did data preprocessing and analyzed data. Yunmeng Chu analyzed the data. Yingfei Ma and Qingshan Jiang supervised this project. All authors have read the final manuscript and approved it for publication.

## CONFLICTS OF INTEREST

The authors declare no conflicts of interest.

## Supporting information

Supplementary information.

Supplementary information.

Supplementary information.

## Data Availability

The datasets and the demo of the NN model are available in the GitHub repository (https://github.com/gsgowell/microbial_markers_identification). Supporting Information Materials (figures, tables, scripts, graphical abstract, slides, videos, Chinese translated version, and update materials) may be found in the online DOI or iMeta Science http://www.imeta.science/.

## References

[1] Tropini, Carolina , Kristen A. Earle , Kerwyn Casey Huang , and Justin L. Sonnenburg . 2017. “The Gut Microbiome: Connecting Spatial Organization to Function.” Cell Host & Microbe 21: 433–42. 10.1016/j.chom.2017.03.010 28407481 PMC5576359

[2] Morgan, Xochitl C. , Timothy L. Tickle , Harry Sokol , Dirk Gevers , Kathryn L. Devaney , Doyle V. Ward , Joshua A. Reyes , et al. 2012. “Dysfunction of the Intestinal Microbiome in Inflammatory Bowel Disease and Treatment.” Genome Biology 13: R79. 10.1186/gb-2012-13-9-r79 23013615 PMC3506950

[3] Cox, Laura M. , and J. Blaser Martin . 2015. “Antibiotics in Early Life and Obesity.” Nature Reviews Endocrinology 11: 182–90. 10.1038/nrendo.2014.210 PMC4487629 25488483

[4] Manrique, Pilar , Benjamin Bolduc , Seth T. Walk , Oost J. der Van , Willem M. de Vos , and Mark J. Young . 2016. “Healthy Human Gut Phageome.” Proceedings of the National Academy of Sciences of the United States of America 113: 10400. 10.1073/pnas.1601060113 27573828 PMC5027468

[5] Segata, Nicola , Jacques Izard , Levi Waldron , Dirk Gevers , Larisa Miropolsky , Wendy S. Garrett , and Curtis Huttenhower . 2011. “Metagenomic Biomarker Discovery and Explanation.” Genome Biology 12: R60. 10.1186/gb-2011-12-6-r60 21702898 PMC3218848

[6] Qin, Junjie , Yingrui Li , Zhiming Cai , Shenghui Li , Jianfeng Zhu , Fan Zhang , Suisha Liang , et al. 2012. “A Metagenome‐Wide Association Study of Gut Microbiota in Type 2 Diabetes.” Nature 490: 55–60. 10.1038/nature11450 23023125

[7] Karlsson, Fredrik H. , Valentina Tremaroli , Intawat Nookaew , Göran Bergström , Carl Johan Behre , Björn Fagerberg , Jens Nielsen , and Fredrik Bäckhed . 2013. “Gut Metagenome in European Women with Normal, Impaired and Diabetic Glucose Control.” Nature 498: 99–103. 10.1038/nature12198 23719380

[8] Nielsen, H. Bjørn , Mathieu Almeida , Agnieszka Sierakowska Juncker , Simon Rasmussen , Junhua Li , Shinchi Sunagawa , Damian R. Plichta , et al. 2014. “Identification and Assembly of Genomes and Genetic Elements in Complex Metagenomic Samples without using Reference Genomes.” Nature Biotechnology 32: 822–8. 10.1038/nbt.2939 24997787

[9] Liu, Ruixin , Hong Jie , Xu Xiaoqiang , Feng Qiang , Dongya Zhang , Yanyun Gu , and Shi Juan , et al. 2017. “Gut Microbiome and Serum Metabolome Alterations in Obesity and after Weight‐Loss Intervention.” Nature Medicine 23: 859–68. 10.1038/nm.4358 28628112

[10] Wen, Chengping , Zhijun Zheng , Shao Tiejuan , Lin Liu , Zhijun Xie , Emmanuelle Le Chatelier , and He Zhixing , et al. 2017. “Quantitative Metagenomics Reveals Unique Gut Microbiome Biomarkers in Ankylosing Spondylitis.” Genome Biology 18: 142. 10.1186/s13059-017-1352-6 28750650 PMC5530561

[11] David, Lawrence A. , Corinne F. Maurice , Rachel N. Carmody , David B. Gootenberg , Julie E. Button , Benjamin E. Wolfe , Alisha V. Ling , et al. 2014. “Diet Rapidly and Reproducibly alters the Human Gut Microbiome.” Nature 505: 559–63. 10.1038/nature12820 24336217 PMC3957428

[12] Yu, Ta Chung , Fangfang Guo , Yanan Yu , Tiantian Sun , Dan Ma , Jixuan Han , Yun Qian , et al. 2017. “Fusobacterium nucleatum Promotes Chemoresistance to Colorectal Cancer by Modulating Autophagy.” Cell 170: 548–63. 10.1016/j.cell.2017.07.008 28753429 PMC5767127

[13] Ilseung, Cho , and Martin J. Blaser . 2012. “The Human Microbiome: At the Interface of Health and Disease.” Nature Reviews Genetics 13: 260–70. 10.1038/nrg3182 PMC3418802 22411464

[14] Koh, Ara , De Vadder Filipe , Petia Kovatcheva‐Datchary , and Fredrik Bäckhed . 2016. “From Dietary Fiber to Host Physiology: Short‐Chain Fatty Acids as Key Bacterial Metabolites.” Cell 165: 1332–45. 10.1016/j.cell.2016.05.041 27259147

[15] Zhao, Liping , Feng Zhang , Xiaoying Ding , Guojun Wu , Yan Y. Lam , Xuejiao Wang , Huaqing Fu , et al. 2018. “Gut Bacteria Selectively Promoted by Dietary Fibers Alleviate Type 2 Diabetes.” Science 359: 1151–6. 10.1126/science.aao5774 29590046

[16] Alipanahi, Babak , Andrew Delong , Matthew T. Weirauch , and Brendan J. Frey . 2015. “Predicting the Sequence Specificities of DNA‐ and RNA‐Binding Proteins by Deep Learning.” Nature Biotechnology 33: 831–8. 10.1038/nbt.3300 26213851

[17] Sung, Jaeyun , Seunghyeon Kim , Josephine Jill T. Cabatbat , Sungho Jang , Yong Su Jin , Gyoo Yeol Jung , Nicholas Chia , and Pan‐Jun Kim . 2017. “Global Metabolic Interaction Network of the Human Gut Microbiota for Context‐Specific Community‐Scale Analysis.” Nature Communications 8: 15393. 10.1038/ncomms15393 PMC5467172 28585563

[18] Ghanat, Bari Mehrab , Choong Yong Ung , Cheng Zhang , Shizhen Zhu , and Hu Li . 2017. “Machine Learning‐Assisted Network Inference Approach to Identify a New Class of Genes that Coordinate the Functionality of Cancer Networks.” Scientific Reports 7: 6993. 10.1038/s41598-017-07481-5 28765560 PMC5539301

[19] Camacho, Diogo M. , Katherine M. Collins , Rani K. Powers , James C. Costello , and James J. Collins . 2018. “Next‐Generation Machine Learning for Biological Networks.” Cell 173: 1581–92. 10.1016/j.cell.2018.05.015 29887378

[20] Angermueller, Christof , Pärnamaa Tanel , Parts Leopold , and Stegle Oliver . 2016. “Deep Learning for Computational Biology.” Molecular Systems Biology 12: 878. 10.15252/msb.20156651 27474269 PMC4965871

[21] Kulmanov, Maxat , Mohammed Asif Khan , and Robert Hoehndorf . 2017. “DeepGO: Predicting Protein Functions from Sequence and Interactions using a Deep Ontology‐Aware Classifier.” Bioinformatics 34: 660–8. 10.1093/bioinformatics/btx624 PMC5860606 29028931

[22] Arango‐Argoty, Gustavo , Emily Garner , Amy Pruden , Lenwood S. Heath , Peter Vikesland , and Liqing Zhang . 2018. “DeepARG: A Deep Learning Approach for Predicting Antibiotic Resistance Genes from Metagenomic Data.” Microbiome 6: 23. 10.1186/s40168-018-0401-z 29391044 PMC5796597

[23] Kermany, Daniel S. , Michael Goldbaum , Wenjia Cai , Carolina C. S. Valentim , Huiying Liang , Sally L. Baxter , and Alex McKeown , et al. 2018. “Identifying Medical Diagnoses and Treatable Diseases by Image‐Based Deep Learning.” Cell 172: 1122‐31. 10.1016/j.cell.2018.02.010 29474911

[24] Chen, Hongming , Ola Engkvist , Yinhai Wang , Marcus Olivecrona , and Thomas Blaschke . 2018. “The Rise of Deep Learning in Drug Discovery.” Drug Discovery Today 23: 1241–50. 10.1016/j.drudis.2018.01.039 29366762

[25] Breiman, Leo . 2001. “Random Forests.” Machine Learning 45: 5–32. 10.1023/A:1010933404324

[26] Nearing, Jacob T. , M. Douglas Gavin , Molly G. Hayes , Jocelyn MacDonald , Dhwani K. Desai , Nicole Allward , and Casey Jones , et al. 2022. “Microbiome Differential Abundance Methods Produce Different Results across 38 Datasets.” Nature Communications 13: 342. 10.1038/s41467-022-28034-z PMC8763921 35039521

[27] Huynhthu, Vân Anh , Irrthum Alexandre , Louis Wehenkel , and Geurts Pierre . 2010. “Inferring Regulatory Networks from Expression Data using Tree‐Based Methods.” PLOS One 5: e12776. 10.1371/journal.pone.0012776 20927193 PMC2946910

[28] Korem, Tal , David Zeevi , Niv Zmora , Omer Weissbrod , Noam Bar , Maya Lotan‐Pompan , and Tali Avnit‐Sagi , et al. 2017. “Bread Affects Clinical Parameters and Induces Gut Microbiome‐Associated Personal Glycemic Responses.” Cell Metabolism 25(6): P1243–53. 10.1016/j.cmet.2017.05.002 28591632

[29] Wescombe, Philip A. , Nicholas C. K. Heng , Jeremy P. Burton , Chris N. Chilcott , and John R. Tagg . 2009. “ *Streptococcal bacteriocins* and the Case for *Streptococcus salivarius* as Model Oral Probiotics.” Future Microbiology 4: 819–35. 10.2217/fmb.09.61 19722837

[30] Hammes, Hans‐Peter , Du Xueliang , Edelstein Diane , Taguchi Tetsuya , Matsumura Takeshi , Qida Ju , and Jihong Lin , et al. 2003. “Benfotiamine Blocks Three Major Pathways of Hyperglycemic Damage and Prevents Experimental Diabetic Retinopathy.” Nature Medicine 9: 294–9. 10.1038/nm834 12592403

[31] Suez, Jotham , Tal Korem , David Zeevi , Gili Zilberman‐Schapira , Christoph A. Thaiss , Ori Maza , and David Israeli , et al. 2014. “Artificial Sweeteners Induce Glucose Intolerance by Altering the Gut Microbiota.” Nature 514: 181–6. 10.1038/nature13793 25231862

[32] Gurung, Manoj , Zhipeng Li , Hannah You , Richard Rodrigues , Donald B. Jump , Andrey Morgun , and Natalia Shulzhenko . 2020. “Role of Gut Microbiota in Type 2 Diabetes Pathophysiology.” EBioMedicine 51: 102590. 10.1016/j.ebiom.2019.11.051 31901868 PMC6948163

[33] Murri, Mora , Isabel Leiva , Juan Miguel Gomezzumaquero , Francisco J. Tinahones , Fernando Cardona , and Federico Soriguer . 2013. “Gut Microbiota in Children with Type 1 Diabetes Differs from that in Healthy Children: A Case‐Control Study.” BMC Medicine 11: 46. 10.1186/1741-7015-11-46 23433344 PMC3621820

[34] Wu, Xiaokang , Chaofeng Ma , Lei Han , Muhammad Nawaz , Fei Gao , Xuyan Zhang , and Pengbo Yu , et al. 2010. “Molecular Characterisation of the Faecal Microbiota in Patients with Type II Diabetes.” Current Microbiology 61: 69–78. 10.1007/s00284-010-9582-9 20087741

[35] Louis, Petra , Georgina L. Hold , and Harry J. Flint . 2014. “The Gut Microbiota, Bacterial Metabolites and Colorectal Cancer.” Nature Reviews Microbiology 12: 661–72. 10.1038/nrmicro3344 25198138

[36] Jandhyala, Sai Manasa , A. Madhulika , G. Deepika , G. Venkat Rao , D. Nageshwar Reddy , Chivukula Subramanyam , and Mitnala Sasikala , et al. 2017. “Altered Intestinal Microbiota in Patients with Chronic Pancreatitis: Implications in Diabetes and Metabolic Abnormalities.” Scientific Reports 7: 43640. 10.1038/srep43640 28255158 PMC5334648

[37] Dewulf, Evelyne M. , D. Cani Patrice , Sandrine P. Claus , Susana Fuentes , Philippe G. B. Puylaert , Audrey M. Neyrinck , and Laure B. Bindels , et al. 2013. “Insight into the Prebiotic Concept: Lessons from an Exploratory, Double Blind Intervention Study with Inulin‐Type Fructans in Obese Women.” Gut 62: 1112–21. 10.1136/gutjnl-2012-303304 23135760 PMC3711491

[38] Segata, Nicola , Levi Waldron , Annalisa Ballarini , Vagheesh Narasimhan , Olivier Jousson , and Curtis Huttenhower . 2012. “Metagenomic Microbial Community Profiling using Unique Clade‐Specific Marker Genes.” Nature Methods 9: 811–4. 10.1038/nmeth.2066 22688413 PMC3443552

[39] Buchfink, Benjamin , Chao Xie , and Daniel H. Huson . 2015. “Fast and Sensitive Protein Alignment using DIAMOND.” Nature Methods 12: 59–60. 10.1038/nmeth.3176 25402007

[40] Guo, Shun , Qingshan Jiang , Lifei Chen , and Donghui Guo . 2016. “Gene Regulatory Network Inference using Pls‐Based Methods.” BMC Bioinformatics 17: 545. 10.1186/s12859-016-1398-6 28031031 PMC5192600

