# Supplementary material for: A neural network‐based framework to understand the type 2 diabetes‐related alteration of the human gut microbiome
Source: Imeta. 2022 May 5;1(2):e20. doi: 10.1002/imt2.20 (PMC10989819; doi:10.1002/imt2.20)
Supplement: Supplementary file 1 — Supplementary information. [file IMT2-1-e20-s003.docx]

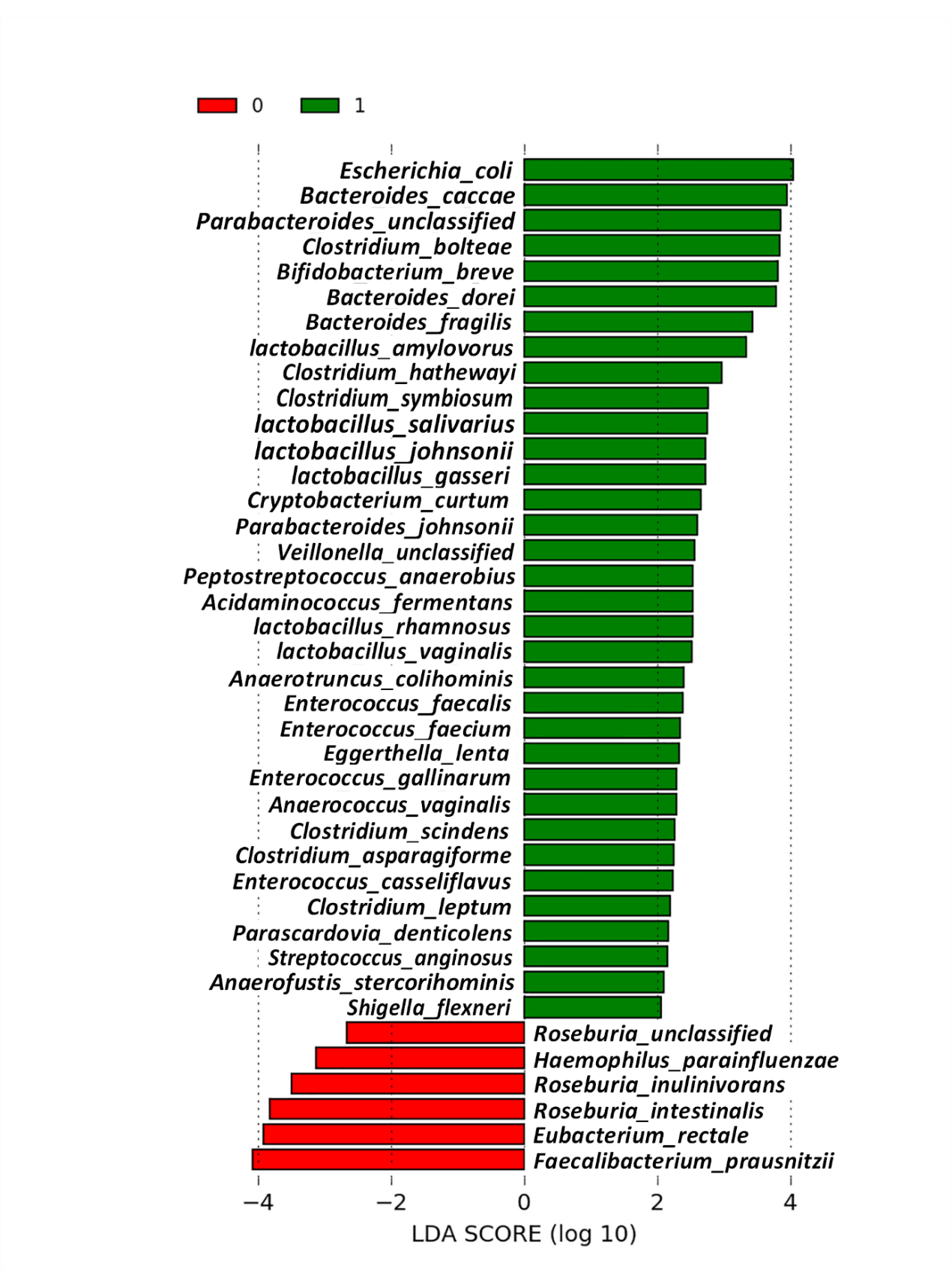


Figure S1. Marker species selected by LEfSe from the species-level profile of D1.





| Figure S2. The functional category of the marker COGs on module or pathway level by using BlastKOALA (51 entries (56.7%) annotated). | |
| --- | --- |
| protein families: genetic information processing | 10 |
| Carbohydrate | 8 |
| Genetic information processing | 6 |
| Unclassified | 6 |
| Unclassified: metabolism | 4 |
| Amino acid metabolism | 3 |
| Metabolism of cofactors and vitamins | 2 |
| Unclassified: signaling and cellular processes | 2 |
| Unclassified: genetic information processing | 2 |
| Cellular processes | 1 |
| Protein families: metabolism | 2 |
| protein families: signaling and cellular processes | 1 |
| Metabolism of terpenoids and polyketides | 1 |
| Glycan biosynthesis and metabolism | 1 |
| Energy metabolism | 1 |
| Lipid metabolism | 1 |
