# Supplementary material for: A neural network‐based framework to understand the type 2 diabetes‐related alteration of the human gut microbiome
Source: Imeta. 2022 May 5;1(2):e20. doi: 10.1002/imt2.20 (PMC10989819; doi:10.1002/imt2.20)
Supplement: Supplementary file 3 — Supplementary information. [file IMT2-1-e20-s002.docx]

The files and the code in this repository correspond to the following manuscript:

A Neural Network-based Framework to Understand the Type 2 Diabetes-related Alteration of the Human Gut Microbiome

Our goal with this work was providing a framework for identifying the disease-related biomarkers. Compared with traditional methods, our method would likely capture some easily ignored biomarkers that varied little across samples.

**Tutorials**

**Data preparing**

Our framework can be applied for all kinds of profile data. In our manuscript, we used MetaPhlAn2.7.4 and Diamond (v0.9.14.115) to obtain the species-level microbial profile and the bacterial gene profile from two datasets (D1, 368 samples, accession number in GenBank: PRJNA422434; D2, 391 samples, accession numbers: PRJEB15179 and PRJEB14155). The corresponding datasets were organized into Matlab format files provided in the data fold of this repository (i.e., d_368.mat). Some details of the data in d_368.mat are as following:

‘**X_368**’: A 368*270 numerical matrix M that represents the species-level microbial profile of D1 (368 is the number of samples in D1, 270 is the number of species, M_ij_ represents the relative abundance of the *j*^th^ species in *i*^th^ sample ).

‘**Y_368**’: A 368*1 numerical matrix that represents the state of all samples in D1 (1: diabetes, 0: healthy).

**‘species_name’**: The 270 species names in D1.

**‘gene_name’**: The 4632 gene names in D1.

‘**X_391**’ : The species-level microbial profile of D2.

‘**Y_391**’: The state of all samples in D2.

‘**X_gene_368**’: The gene profile of D1.

We also provided two trained model (**‘diabet_identify_species_d1_minus_d2_plus.h5’** and **‘diabet_identify_gene_d1_minus_d2_plus.h5’**; trained on D1- for testing on D2+ using our markers.

**Random Forest feature ranking**

As for the species-level microbial profile, each species can be viewed as the feature of the samples. And it is similar to the gene profile (i.e., each gene can be viewed as the feature of the samples). In our framework, we firstly used Random Forest method to calculated the importance of each feature (i.e., species or gene) in the profile data. In our study, we used the Matlab package ‘RF_Class_C’ for the relevant calculation. Of cause, other Random Forest tools can also be applied for this step, such as Python RandomForestClassifier. Specifically, as for the D1 species-level profile, ‘X_368’ and ‘Y_368’ are **the input** of the feature ranking step. The importance scores of the 270 species in the profile can be obtained by implementing the Random Forest tool. Then, we reordered the species in ‘X_368’ via the importance scores to construct a new numerical matrix ‘X_368_rf’, which is also provided in d_368.mat. Some details for the feature ranking data are as following:

**‘X_368_rf’ :** A 368*270 numerical matrix M’ that represents the species-level microbial profile of D1 with Random Forest feature ranking. The first column of M’ represents the relative abundances of the species with most importance score for all samples, while the last column of M’ represents that with least importance score for all samples.

**‘X_gene_rf’:** The gene profile of D1 with Random Forest feature ranking.

**‘X_391_rf’:** The species-level microbial profile of D2 with Random Forest feature ranking.

**Neural Network (NN) classifying for identifying the biomarkers**

In this step, the NN model was applied for determining how many features should be selected. That is, the profile data with different number of features were feed to the NN classifier respectively, and the one with best classification performance was the final result. The python code of this step is provided in the demo fold of this repository (i.e., **main_kfold.py**). The input of the this step is the feature ranked profile data and the corresponding state data. For example, the dataset[‘X_368_rf’][:, 0:10] represents the ranked profile data with first 10 features. Together with dataset[‘Y_368’] as the input, by implementing the code, we can obtain the performance of the classifier (e.g., NN) with the 10 features.

**Regression analyzing**

The python code of this step is provided in the demo fold of this repository (i.e., **regression_kfold.py**). The input of the this step is the profile data with the selected features (e.g., dataset[‘X_368_rf’][:, 0:40])and the corresponding T2D-related factors (e.g., dataset[‘Y_fbg’]).
